# Epidemiology of Epstein-Barr virus infection and infectious mononucleosis in the United Kingdom

**DOI:** 10.1186/s12889-020-09049-x

**Published:** 2020-06-12

**Authors:** Ashvin Kuri, Benjamin Meir Jacobs, Nikki Vickaryous, Julia Pakpoor, Jaap Middeldorp, Gavin Giovannoni, Ruth Dobson

**Affiliations:** 1 Preventive Neurology Unit, Wolfson Institute of Preventive Medicine, Queen Mary University London, London, UK; 2 Department of Neurology, Royal London Hospital, Barts Health NHS Trust, London, UK; 3 Unit of Health-Care Epidemiology, Nuffield Department of Population Health, University of Oxford, Oxford, UK; 4 Department of Pathology, VU Medical Center, Amsterdam, The Netherlands

**Keywords:** Epstein Barr virus, Infectious mononucleosis, Glandular fever, Seroprevalence, Antibody response

## Abstract

**Background:**

Epstein-Barr Virus (EBV) is a ubiquitous gamma-herpesvirus with which ~ 95% of the healthy population is infected. EBV infection has been implicated in a range of haematological malignancies and autoimmune diseases. Delayed primary EBV infection increases the risk of subsequent complications. Contemporaneous seroepidemiological data is needed to establish best approaches for successful vaccination strategies in the future.

**Methods:**

We conducted a sero-epidemiological survey using serum samples from 2325 individuals between 0 and 25 years old to assess prevalence of detectable anti-EBV antibodies. Second, we conducted a retrospective review of Hospital Episode Statistics to examine changes in Infectious Mononucleosis (IM) incidence over time. We then conducted a large case-control study of 6306 prevalent IM cases and 1,009,971 unmatched controls extracted from an East London GP database to determine exposures associated with IM.

**Results:**

1982/2325 individuals (85.3%) were EBV seropositive. EBV seropositivity increased more rapidly in females than males during adolescence (age 10–15). Between 2002 and 2013, the incidence of IM (derived from hospital admissions data) increased. Exposures associated with an increased risk of IM were lower BMI, White ethnicity, and not smoking.

**Conclusions:**

We report that overall EBV seroprevalence in the UK appears to have increased, and that a sharp increase in EBV seropositivity is seen in adolescent females, but not males. The incidence of IM requiring hospitalisation is increasing. Exposures associated with prevalent IM in a diverse population include white ethnicity, lower BMI, and never-smoking, and these exposures interact with each other. Lastly, we provide pilot evidence suggesting that antibody responses to vaccine and commonly encountered pathogens do not appear to be diminished among EBV-seronegative individuals. Our findings could help to inform vaccine study designs in efforts to prevent IM and late complications of EBV infection, such as Multiple Sclerosis.

## Background

Epstein-Barr Virus (EBV) is a ubiquitous gamma-herpesvirus with which ~ 95% of healthy adults are infected [1]. EBV is the commonest causative agent of Infectious Mononucleosis (IM), a triad of pharyngitis, lymphadenopathy and fever in the context of acute EBV infection [2]. EBV infection is also implicated in a range of haematological malignancies (Burkitt’s lymphoma, gastric lymphoma, and Non-Hodgkin lymphoma), and has been strongly associated with autoimmune diseases such as Multiple Sclerosis (MS).

EBV seroprevalence increases with age, and tends to be higher among females, non-Caucasian ethnic groups, and people living in socio-economically deprived households [3, 4]. Later age at EBV infection confers both a higher risk of IM and a higher risk of severe features [2]. Overall EBV seroprevalence rates among children and adolescents appear to be decreasing in some populations, implying that the population at risk of complicated primary EBV infection may be increasing [4–6].

Various strands of evidence suggest a pathogenic role for EBV infection in MS: EBV seronegativity is exceptionally rare in people with MS, EBV seropositivity is higher in people with MS, higher anti-EBV antibody titres are associated with increased risk of MS, and prior IM increases the risk of subsequent MS [7–9]. Understanding the epidemiology of EBV infection during childhood and adolescence is essential [10], for both maximising the efficacy and safety of vaccine studies [11], and in order to inform power calculations for future potential interventional studies [11].

Previous vaccine studies have demonstrated efficacy against symptomatic IM but not EBV seroconversion [12]; however multivalent vaccines are currently being developed with the aim of preventing seroconversion [13]. Alongside this, there remains an urgent need to better understand the current epidemiology of EBV transmission, and potential associations with both early and late seroconversion.

We therefore set out to update and establish current EBV seroprevalence across the UK, establish the potential effect of any change in the pattern of UK EBV seroprevalence on prevalence of hospital admissions with Infectious Mononucleosis (IM), and examine predictors of late EBV infection (IM) in a large primary care cohort. Finally, we used a small cohort of truly EBV-negative samples from adults to explore whether EBV seronegativity is associated with a reduced immune response to other vaccine-preventable and/or ubiquitous infections.

## Methods

### Samples

Serum samples used to investigate EBV seroprevalence across the UK were requested from Public Health England Seroepidemiology Unit (SEU). The SEU houses a serum repository curated from residual samples from NHS laboratories in England. Anonymised samples retain data pertaining to age, sex, date and laboratory of collection. Two thousand five hundred samples spread across age groups and English geographical area (London, South West, West Midlands, North West, North East) collected in 2016 were requested; 2366 samples were available for analysis.

A second cohort of 21 adult EBV-seronegative and 42 age and gender matched EBV-seropositive samples from a Netherlands cohort were used to compare the immunological properties associated with serostatus. EBV seronegativity was defined using an in-house VCA-IgG and EBNA1-IgG ELISA, followed by confirmation with an IgG immunoblot against VCA, EA, and EBNA [14].

All serum samples were stored at -80C until analysis, and freeze thaw cycles kept to a minimum. No samples had > 3 freeze thaw cycles prior to analysis.

### Enzyme-linked immunosorbent assay (ELISA)

The anti-Epstein Barr virus (EBV-VCA) IgG Human ELISA kit (Abcam, cat no. ab108730) was used for qualitative determination of IgG antibodies against EBV viral capsid antigen (VCA) in human serum. IgG VCA antibodies peak two to 4 weeks post EBV infection, and persist for life, serving as a marker for recent or historic EBV infection. Serum samples were diluted 1:100 immediately prior to use and assayed according to the manufacturer’s protocol. Positive, negative and cut-off controls were run in duplicate; samples were run in singlicate. Samples falling within the +/− 10% cut-off range were repeated in duplicate; samples remaining in the +/− 10% cut-off range were not included in the final analysis.

Quantitative measurement of IgG directed against *Rubella* and *Varicella zoster virus* (VZV) and semi-quantitative measurement of IgG against *Bordetella pertussis* were performed in duplicate using ELISA kits (cat no. KA0223, KA1456, and KA2090 respectively; Abnova Corporation, Taipei City, Taiwan) according to manufacturer’s instructions. Assay validity was confirmed using control samples provided by the manufacturer in all cases.

### Hospital episode statistics (HES)

Hospital admissions where infectious mononucleosis (IM) was recorded in healthcare notes as either a primary or secondary diagnostic code during admission were examined. Hospital Episode Statistics (HES) incorporate every episode of hospital day-case or overnight inpatient care in National Health Service (NHS) hospitals [15]. Record-linkage was performed in order to construct absolute numbers of recorded hospital attendances with IM for each year 2002–2013. Infectious mononucleosis was defined as ICD-10 code B27.9 and ICD-9 code 075. Absolute numbers of cases of IM per age band were converted to rates per 100,000 person-years for each 5-year age band using mid-year population estimates from Office for National Statistics (ONS) as the denominator.

### Primary care data analysis

The East London Primary Care dataset consists of de-identified primary care (General Practitioner, GP) healthcare records from all GP practices using the EMIS electronic healthcare records system (https://www.emishealth.com/) across four clinical commissioning groups (CCGs) in east London - Hackney, Newham, Tower Hamlets and Waltham Forest.

To determine environmental and ethnic exposures associated with IM risk, we conducted a large case-control study using East London GP database records. Demographic details for all participants within the database were extracted, including self-declared ethnicity according to ONS codes at GP registration. Demographic details were defined as previously described [16]. Age was defined at age data extraction (1st February 2018), and deprivation according to IMD raw scores for each lower layer super output area (LSOA).

Infectious mononucleosis was defined using codes for ‘Glandular fever’, ‘confirmed glandular fever’, ‘gammaherpesviral mononucleosis’, ‘infective mononucleosis’, ‘infectious mononucleosis’, and ‘Pfeiffer’s disease’.

### Ethical approval

Public Health England Seroepidemiology Unit (SEU) sample analysis was in line with SEU ethical approvals (reference 05/Q0505/45); analysis of the EBV seronegative cohort was covered by internal ethical approval from VU University, Amsterdam. Primary and secondary care data analysis was performed on fully anonymised unlinked records and separate ethical approvals were not required.

### Statistical analysis

Determinants of EBV serostatus and IM were examined using univariable and multivariable logistic regression. The strength of association between a variable and EBV serostatus was determined using the likelihood ratio of the full model vs that containing only confounders. Differential proportions of seropositivity to other infections was compared between EBV-positive and EBV-negative cohorts using Fisher’s exact test. Unless otherwise stated, results are presented as estimate followed by 95% confidence interval (CI). All analyses were conducted using R version 3.6.1 in RStudio.

## Results

### UK EBV seroprevalence

**Fig. 1:**
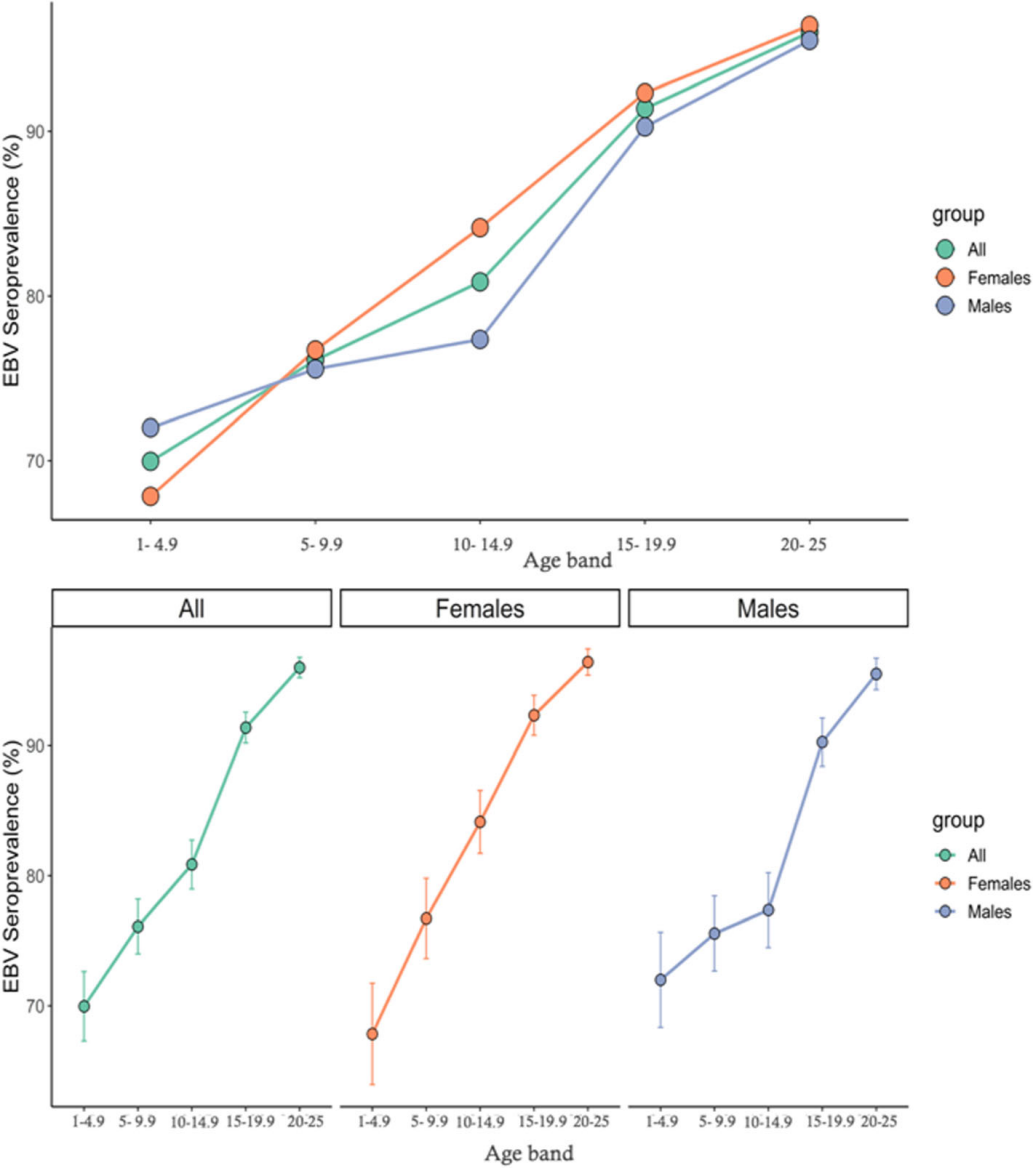
Top panel – EBV seroprevalence among healthy volunteers split by sex and age range. Bottom panel – same data as top panel, presented separately with 95% confidence intervals for each sex

41/2366 individuals had indeterminate EBV serology results and were excluded from further analysis. Of the 2325 participants with definite serostatus results, there was a roughly equal gender split (51.4% female). Sample demographics are displayed in supplementary table 1. Overall 1982/2325 (85.3%) were EBV seropositive. EBV seroprevalence increased with age from 1 to 4.9 (67.8% females, 72.0% males seropositive) to 20–25 (96.4% females, 95.5% males seropositive, Table 1, Fig. 1). Each year increase in age corresponded to a 12% increase in the odds of seropositivity (OR = 1.12, 95%CI 1.10–1.14, *p* < 0.0001). Region was associated with EBV seroprevalence in a univariable model, with evidence of lower seropositivity in non-London regions (*p* = 0.0059), however this effect dissipated on correction for age and sex (data not shown).

**Table 1:** Raw numbers of seropositive and seronegative individuals within each age and sex category, univariate and multivariable odds ratios for seropositivity for males, females, and combined estimates

Age band	Females				Males				Overall	
	Negative (n)	Positive (n)	% seropositive	OR (95%CI)	Negative (n)	Positive (n)	% seropositive	OR (95%CI)	Univariable (OR; 95%CI)	Multivariable^**a**^ (OR; 95%CI)
0–4.9	46	97	67.8	1 (reference)	42	108	72.0	1 (reference)	1 (reference)	1 (reference)
5–9.9	44	145	76.7	1.56 (0.96–2.55)	54	167	75.6	1.20 (0.75–1.92)	1.37 (0.97–1.77)	1.38 (0.99–1.94)
10–14.9	36	191	84.1	2.52 (1.53–4.17)	48	164	77.4	1.33 (0.82–2.15)	1.81 (1.29–2.33)	1.81 (1.28–2.56)
15–19.9	23	277	92.3	5.71 (3.32–10.06)	25	232	90.3	3.61 (2.11–6.30)	4.55 (3.10–6.00)	4.52 (3.08–6.71)
20–25	12	324	96.4	12.80 (6.72–26.20)	13	277	95.5	8.29 (4.39–16.63)	10.32 (6.54–14.10)	9.93 (6.27–16.28)

^**a**^ multivariable OR includes correction for region of sample origin and sex

Seroprevalence estimates were similar between males and females in most age groups other than the 10–14.9 age band (Fig. 1). At age 10–14.9, the EBV seroprevalence point estimate was higher in females (84.1, 95%CI 79.4–88.9%) than males (77.4, 95%CI 71.0–83.0%), although these estimates had relatively wide and overlapping confidence intervals.

### Temporal trends in the incidence of infectious mononucleosis 2002–2013

**Fig. 2:**
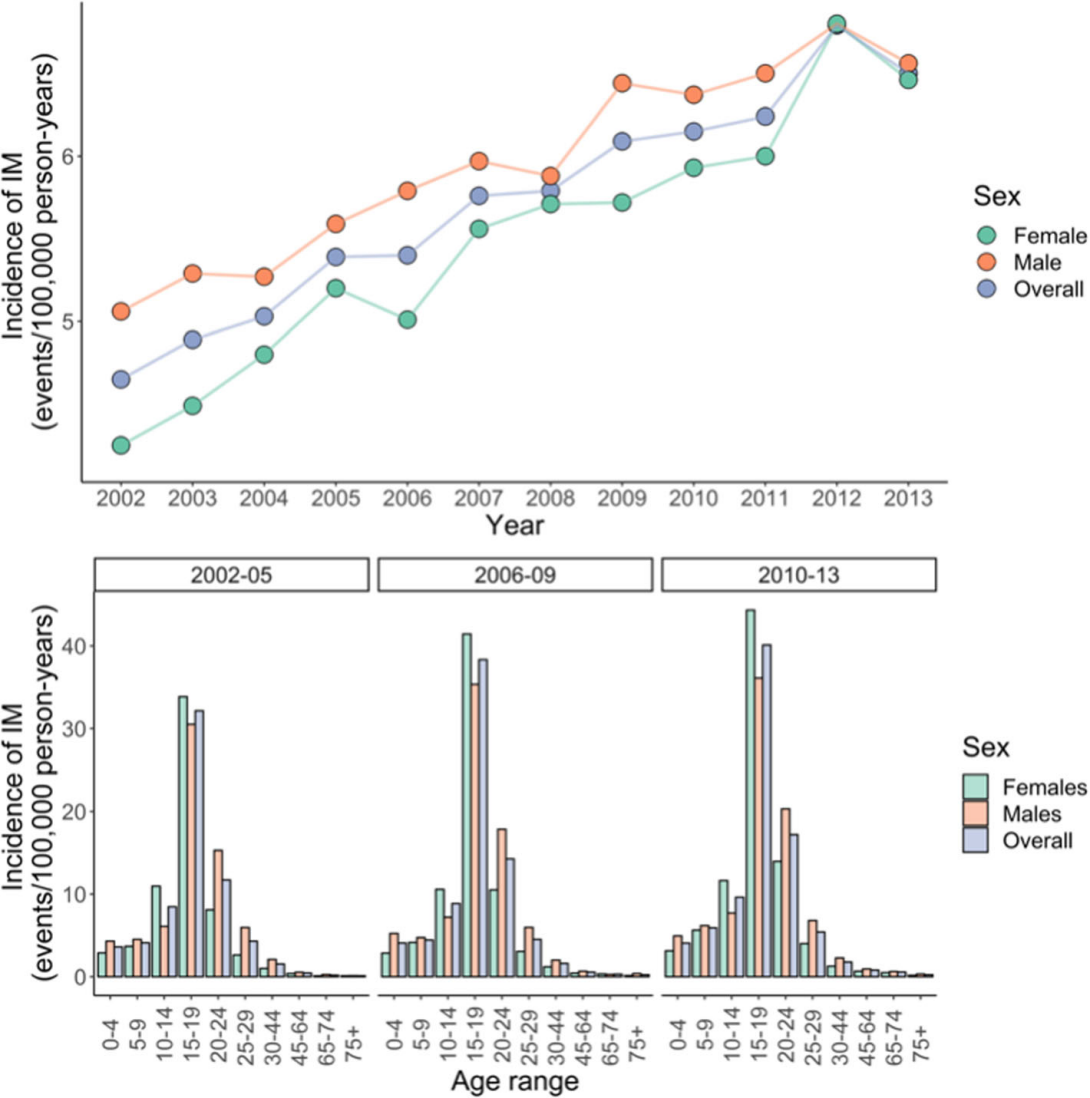
Top panel – incidence of infectious mononucleosis (IM) derived from Hospital Episode Statistics between 2002 and 2013 divided by sex. Bottom panel – same data as top panel presented grouped by epochs of 3 years, presented separately for each age range

Between 2002 and 2013, the incidence of hospital admissions (per 100,000 patient years) associated with a coded diagnosis of IM steadily rose for males and females (supplementary table 2). Overall, the incidence of hospital admission associated with IM was higher in males than females, however for individuals age 10–15, incidence was higher in females than males. There was no clear shift in sex ratio or the age distribution of incident IM cases, with those aged 15–19 years accounting for the majority of diagnoses in both time periods studies (Fig. 2).

### Exposures associated with infectious mononucleosis

Data were available for 6306 IM cases and 1,009,971 unmatched controls. Demographics of included participants are shown in Table 2. We fitted multivariable logistic regression models using prevalent IM status (i.e. any historical episode of IM) as the outcome, controlling for sex and age at the time of data extraction. Adding BMI, ethnicity, smoking, and IMD decile to the model all improved the model fit, suggesting that these exposures explain at least some of the variation in IM prevalence.

**Table 2:** Demographics of IM cases and unmatched controls in the East London GP database, including prevalence of IM among each group

	Group	Controls (*n* = 1,009,971)	Cases (*n* = 6306)	Prevalence (% cases per group)
Age (mean; SD)		40.49 (15.44)	39.19 (13.08)	
Sex	Female	493,384	3193	0.64
	Male	516,573	3113	0.60
Ethnicity	White	440,066	4865	1.09
	Black	135,689	282	0.21
	Other	114,712	283	0.25
	S.Asian	217,426	377	0.17
BMI	Normal	388,300	3031	0.77
	Obese	130,458	543	0.41
	Overweight	253,323	1361	0.53
	Underweight	136,876	966	0.70
Smoking	Never smoker	561,648	3396	0.60
	Ever smoker	399,975	2800	0.70
IMD decile	1 (most deprived	137,431	561	0.41
	2	317,377	1692	0.53
	3	303,976	1746	0.57
	4	120,676	924	0.76
	5	55,535	628	1.12
	6	20,756	244	1.16
	7	13,018	129	0.98
	8	6154	75	1.20
	9	6532	89	1.34
	10 (least deprived)	335	8	2.33

**Fig. 3:**
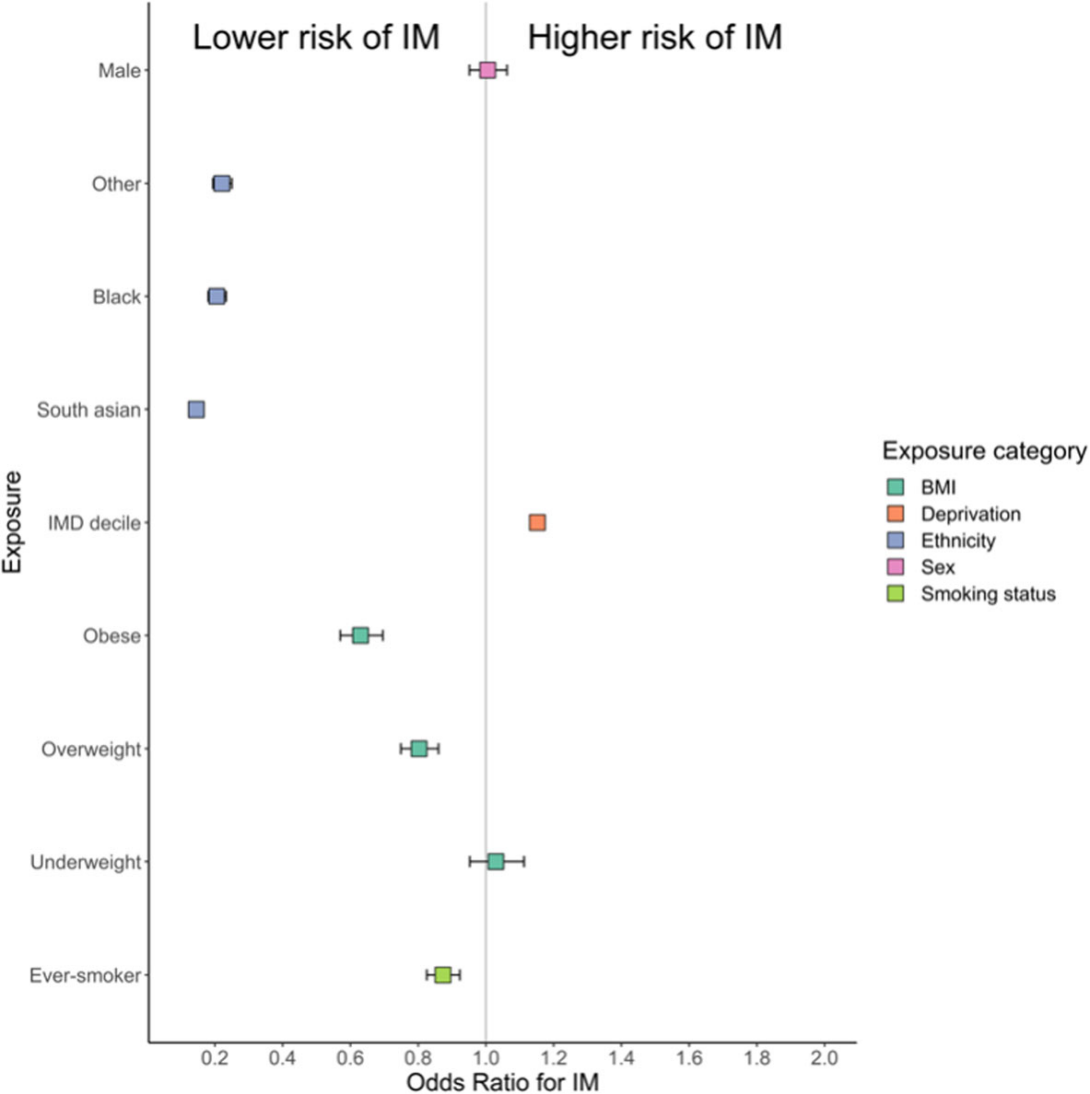
Forest plot depicting exposure associations with IM in the East London GP database. Data points represent odds ratios +/− 95% confidence intervals

The effects associated with these factors persisted in a combined multivariable model incorporating all factors. Factors with an apparent protective effect with respect to IM in this model were elevated BMI (Overweight [OR = 0.80, 95%CI 0.75–0.86], obese [OR = 0.63, 95%CI 0.57–0.70]; normal BMI as reference category), non-white ethnicity [Black OR = 0.21, 95%CI 0.18–0.23, Asian OR = 0.14, 95%CI 0.13–0.16, Other ethnicity OR = 0.22, 95%CI 0.19–0.25]; White ethnicity as reference category), and a history of ever smoking (OR = 0.87, 95%CI 0.83–0.92; never smoking as reference category). Increased IMD decile (i.e. less deprived LSOA) was associated with a higher risk of IM (per increase in IMD decile OR = 1.15, 95%CI 1.13–1.17, Fig. 3 and Table 3).

**Table 3:** Multivariable odds ratios for IM with 95% CIs (adjusted for age and sex) derived from East London GP database. *P* values represent empirical p values for each level of the predictor (3rd column) and likelihood ratio p values (4th column) represent the additional improvement in model fit on adding the predictor to a null model consisting of only age and sex

Exposure	OR (95% CI)	*P* value	Likelihood ratio P
Age	0.99 (0.99–0.99)	< 0.0001	
Sex			
Female	1 (reference)		
Male	1.01 (0.95–1.06)	0.85	
Ethnicity			< 0.0001
White	1 (reference)		
Black	0.21 (0.18–0.23)	< 0.0001	
South Asian	0.14 (0.13–0.16)	0.00012	
Other	0.22 (0.19–0.25)	0.00013	
IMD Decile (2015)	1.15 (1.13–1.17)	< 0.0001	< 0.0001
Weight			< 0.0001
Normal weight	1 (reference)		
Obese	0.63 (0.57–0.7)	< 0.0001	
Overweight	0.8 (0.75–0.86)	< 0.0001	
Underweight	1.03 (0.95–1.11)	0.465	
Smoking status			< 0.0001
Never smoker	1 (reference)		
Ever smoker	0.87 (0.83–0.92)	< 0.0001	

We then looked for first-order interactions between all significant predictors of IM status, after controlling for age and gender. We considered an interaction significant if including the interaction term improved model fit at an adjusted p threshold of 0.05/6 = 0.00833. We observed significant pairwise interactions between ethnicity and IMD, ethnicity and weight, ethnicity and smoking, and weight and IMD (supplementary table3). Repeating this analysis using weight as a binary measure (grouping “normal” and “underweight” individuals, and grouping “overweight” and “obese” individuals) gave similar results (data not shown). Strikingly, we noted opposite effects of smoking and body weight depending on ethnic group: the ‘protective’ effect of being overweight was reversed in Asian individuals, and the ‘protective’ effect of smoking was only observed in White individuals.

### EBV seronegativity is not associated with diminished serologic response to vaccine antigens

In order to test the hypothesis that EBV-seropositivity is biologically necessary to prime and maintain B cell memory responses to vaccine antigens, we assayed IgG against recall antigens. Sera were confirmed to be EBV-seronegative using the methods described above; matched control sera were EBV seropositive. We found no evidence of differences in Rubella, Pertussis, or Varicella serostatus between EBV seronegative and EBV seropositive individuals (Supplementary table 4).

## Discussion

In this study we demonstrate the temporal trends in EBV seroprevalence during childhood and adolescence among healthy UK volunteers, and explore socio-demographic associations with IM (a marker of late infection). The seroprevalence estimates in all age bands in our study are higher than in other previous similar studies; additionally, we show that EBV seroprevalence differs between genders in adolescence (10–14.9 years), with male sex conferring a slightly lower risk of EBV seropositivity at this age. This corresponds to a gender imbalance among hospital admissions associated with a code indicating infectious mononucleosis in this age band. Supporting this finding is previous work demonstrating that the prevalence of EBV DNA in healthy tonsils increased with age for males (peak 77.4% for > 35 year-olds), but reached a peak at 79.3% for females from 15 to 24, and remained at that plateau [17]. The implication of these findings is a gender imbalance in EBV seroconversion hazard rates by age, as infectious mononucleosis rates are a function of the interaction between the population at risk (EBV naïve) and EBV-positive population pool [10].

An important finding of our work is the observation that EBV-seronegative individuals do not appear to have lower levels of circulating antibody to common vaccine antigens and pathogens. A major concern regarding EBV vaccines is disruption of the host-pathogen interaction with unintended deleterious consequences for host and population immunity. EBV achieves long-term immune evasion by transforming naive B cells into a resting memory B cell phenotype and activating latency programmes. Given this tropism, and the widespread prevalence of EBV, there is an evolutionary argument that humans have co-evolved with EBV due to a selective advantage offered by the virus. Specifically, it is possible that EBV infection promotes herd and individual immunity to a broad range of pathogens by expanding and maintaining the size of the memory B cell pool. Thus, there is a theoretical concern that vaccination against EBV may have unintended consequences for B cell memory at an individual and population level. Our pilot data is reassuring, and will need to be replicated on a larger scale in order to allay this theoretical concern.

We show that, as would be expected, the seroprevalence of EBV increases with age from 69.9% in the 1–4.9 age bracket to 96.0% among 20–25 year olds. Our results are consistent with previous studies on the determinants of EBV seropositivity, and fit with previous models of EBV infection and seroconversion [10]. Several studies in other populations have demonstrated a decline in age-adjusted seroprevalence of EBV over the past 15 years [4–6]. In contrast, our study demonstrated consistently higher EBV seroprevalence among all age brackets compared to a comparable older study [18] – this may reflect a genuine increase in seroprevalence in the UK, differences in population sampling, or different sensitivity and specificity of the assays used (our study used VCA whereas previous studies have used EBNA). Reassuringly, our estimates closely mirror those from a recent random sampling of UK Biobank participants: among those aged 40–69 years, EBV seroprevalence was 94.7%^1^.

We show that exposures associated with increased IM risk (i.e. late infection) include White ethnicity, normal/low body weight, lower deprivation, and never smoking, in keeping with international data [3, 4]. We found additional evidence of interaction between smoking, body weight, and ethnicity in determining IM risk. We cannot rule out that the exposures we identify as being associated with IM are in fact all proxies for lower levels of household crowding or other early life determinants. Our study used prevalent cases, which introduces the problems of reverse causation and confounding. The finding that effects differ between ethnicities is intriguing. These traits may be proxies for different exposures between different ethnicities (e.g. theoretically, being overweight could be a marker of high wealth in one group and deprivation in another). Alternatively, this may reflect a genuine biological effect whereby BMI and smoking interact with genetic background to influence IM risk.

The use of healthcare databases to study IM is challenging – coded diagnoses of IM are usually not linked to serological evidence, and certainly include non-EBV related infections. Others have used healthcare databases to examine IM incidence [10, 19], highlighting the role of transmission within families [19]. The observed similarities in gender variation in IM and seroconversion during teenage years between HES and serum data is compelling, as is the similarity in admission incidence by age between the UK and Denmark [19]. It must be noted that within the HES dataset the incidence of all diseases appears to be increasing due to improved coding; we have mitigated this to some degree by focussing on recent decades. However, the increase in incidence within the HES dataset is striking, with almost a 50% increase over 10 years.

Understanding the temporal trends and host determinants of EBV serostatus are essential for rational vaccine design and deployment. In principle, vaccination against EBV could help to reduce rates of EBV-associated malignancies and EBV-associated autoimmune diseases. Although there is still no effective vaccine available, several are in development, and phase 2 results from a recombinant gp350 vaccine suggested an effect on IM rates without affecting overall seroprevalence [12]. The population-level efficacy of such a vaccine depends crucially on the age at which it is administered, with earlier vaccines predicted to offer greater overall reductions in rates of IM and other EBV-related sequlae [20]. Our empirical data showing high seroprevalence even in the youngest age bracket support the modelling data, suggesting that, in terms of maximising efficacy, EBV vaccines should be delivered in the first few years of life.

In conclusion, our data suggest that, in the UK, EBV seroconversion is taking place earlier in life, that overall EBV seroprevalence remains very high (> 95% of 21–25 year olds), IM incidence is increasing, and there are several environmental exposures associated with IM risk (ethnicity, deprivation, smoking, and BMI). Furthermore, we provide preliminary evidence that EBV seronegative persons do not have lower levels of circulating antibodies to common pathogens and vaccine antigens, which mitigates some concerns around early life vaccination.

## Supplementary information

**Additional file 1: Table S1.** demographics of EBV-seropositive and EBV-seronegative individuals included in the seroprevalence study. **Table S2.** raw incidence of HES-coded IM (cases per 100,000 person-years) among males and females in three time windows. **Table S3.** likelihood ratio p values comparing models of IM risk without pairwise interaction and with first-order interaction between the named exposures. P values below the Bonferroni-adjusted threshold of 0.008 are highlighted in bold. **Table S4.** Serostatus proportions for vaccine preventable and commonly acquired illnesses between EBV seropositive and EBV seronegative individuals.

## Data Availability

SEU samples and HES data are available to appropriately qualified investigators on application to the relevant bodies in line with their ethical approvals. Permission to use SEU samples can be obtained on application to the SUE steering committee. De-identified East London Primary Care records are available on application by appropriately qualified investigators via the Clinical Effectiveness Group at QMUL. If further details are required, these can be obtained via reasonable request to the corresponding author who will direct enquires to the relevant data custodians and/or steering groups.

## Abbreviations

EBV: Epstein Barr virus
IM: Infectious mononucleosis
SEU: Seroepidemiology unit
HES: Hospital Episode Statistics
GP: General Practice
BMI: Body Mass Index
MS: Multiple Sclerosis
VCA: Viral capsid antigen
EBNA-1: Epstein Barr nuclear antigen-1
ELISA: Enzyme linked immunosorbent assay
VZV: Varicella zoster virus
NHS: National Health Service
ONS: Office for National Statistics
