# Supplementary material for: Epidemiology of Epstein-Barr virus infection and infectious mononucleosis in the United Kingdom
Source: BMC Public Health. 2020 Jun 12;20:912. doi: 10.1186/s12889-020-09049-x (PMC7291753; doi:10.1186/s12889-020-09049-x)
Supplement: Supplementary file 1 — Additional file 1: Table S1. demographics of EBV-seropositive and EBV-seronegative individuals included in the seroprevalence study. Table S2. raw incidence of HES-coded IM (cases per 100,000 person-years) among males and females in three time windows. Table S3. likelihood ratio p values comparing models of IM risk without pairwise interaction and with first-order interaction between the named exposures. P values below the Bonferroni-adjusted threshold of 0.008 are highlighted in bold. Table S4. Serostatus proportions for vaccine preventable and commonly acquired illnesses between EBV seropositive and EBV seronegative individuals. [file 12889_2020_9049_MOESM1_ESM.docx]

Supplementary table 1: demographics of EBV-seropositive and EBV-seronegative individuals included in the seroprevalence study.

|  |  | Negative (n, %) | Positive (n, %) |
| --- | --- | --- | --- |
| Overall n |  | 343 (14.8) | 1982 (85.3) |
| Age band | 1 – 4.9 | 88 (30.0) | 205 (70.0) |
|  | 5 – 9.9 | 98 (23.9) | 312 (76.1) |
|  | 10 – 14.9 | 84 (19.1) | 355 (80.9) |
|  | 15 – 19.9 | 48 (8.6) | 509 (91.4) |
|  | 20 – 25 | 25 (4.0) | 601 (96.0) |
| Sex | F | 161 (13.5) | 1034 (86.5) |
|  | M | 182 (16.1) | 948 (83.9) |
| Location | Exeter | 33 (15.7) | 177 (84.3) |
|  | Manchester | 275 (15.6) | 1487 (84.4) |
|  | Newcastle | 34 (11.0) | 275 (89.0) |
|  | Royal London | 1 (2.3) | 43 (97.7) |

Supplementary table 2: raw incidence of HES-coded IM (cases per 100,000 person-years) among males and females in three time windows.

|  | Males | | | Females | | | F:M ratio | | |
| --- | --- | --- | --- | --- | --- | --- | --- | --- | --- |
|  | **2002-05** | **2006-09** | **2010-13** | **2002-05** | **2006-09** | **2010-13** | **2002-05** | **2006-09** | **2010-13** |
| 0-4 | 4.31 | 5.23 | 4.94 | 2.88 | 2.85 | 3.14 | 0.67 | 0.54 | 0.64 |
| 5-9 | 4.52 | 4.73 | 6.19 | 3.67 | 4.14 | 5.64 | 0.81 | 0.88 | 0.91 |
| 10-14 | 6.09 | 7.2 | 7.7 | 10.96 | 10.58 | 11.64 | 1.80 | 1.47 | 1.51 |
| 15-19 | 30.51 | 35.34 | 36.1 | 33.85 | 41.44 | 44.34 | 1.11 | 1.17 | 1.23 |
| 20-24 | 15.29 | 17.85 | 20.32 | 8.09 | 10.5 | 13.95 | 0.53 | 0.59 | 0.69 |
| 25-29 | 5.96 | 5.97 | 6.8 | 2.63 | 3.05 | 4.01 | 0.44 | 0.51 | 0.59 |
| 30-44 | 2.09 | 2.01 | 2.27 | 1 | 1.21 | 1.27 | 0.48 | 0.60 | 0.56 |
| 45-64 | 0.53 | 0.67 | 0.93 | 0.38 | 0.44 | 0.67 | 0.72 | 0.66 | 0.72 |
| 65-74 | 0.27 | 0.28 | 0.63 | 0.1 | 0.33 | 0.49 | 0.37 | 1.18 | 0.78 |
| 75+ | 0.13 | 0.39 | 0.32 | 0.12 | 0.15 | 0.17 | 0.92 | 0.38 | 0.53 |

|  | BMI | Smoking | IMD | Ethnicity |
| --- | --- | --- | --- | --- |
| BMI |  |  |  |  |
| Smoking | 0.421 |  |  |  |
| IMD | **<0.005** | 0.112 |  |  |
| Ethnicity | **<0.0001** | **<0.0001** | **<0.0001** |  |

Supplementary table 3: likelihood ratio p values comparing models of IM risk without pairwise interaction and with first-order interaction between the named exposures. P values below the Bonferroni-adjusted threshold of 0.008 are highlighted in bold.

Supplementary table 4: Serostatus proportions for vaccine preventable and commonly acquired illnesses between EBV seropositive and EBV seronegative individuals

1. **Rubella**

|  | Rubella Positive | Rubella Negative | Totals |
| --- | --- | --- | --- |
| EBV Seropositive | 37 | 5 | 42 |
| EBV Seronegative | 19 | 2 | 21 |

1. **VZV**

|  | VZV Positive | VZV Negative | Totals |
| --- | --- | --- | --- |
| EBV Seropositive | 38 | 4 | 42 |
| EBV Seronegative | 18 | 3 | 21 |

**(c) Pertussis**

|  | Pertussis Positive | Pertussis Negative | Totals |
| --- | --- | --- | --- |
| EBV Seropositive | 25 | 17 | 42 |
| EBV Seronegative | 9 | 12 | 21 |
